# Understanding Body Perception Distortions in Complex Regional Pain Syndrome

**DOI:** 10.1002/ejp.70418

**Published:** 2026-10-12

**Authors:** Matthew R. Longo

**Affiliations:** ^1^ School of Psychological Sciences, Birkbeck University of London London UK

This journal recently published a paper by Naylor et al. (2026) entitled ‘Variations in current and desired limb perception in complex regional pain syndrome’. Misperceptions and delusions about one's body are a striking feature of many neurological and psychiatric conditions, from phantom limbs in amputees to body image distortions in eating disorders. Pain is no exception, and misperceptions of the body have been reported in several varieties of chronic pain, notably including complex regional pain syndrome (CRPS; Lewis et al. 2007).

In the present study, Naylor et al. (2026) administered an exploratory online survey to 115 individuals diagnosed with CRPS asking them about both their current perceptions of their affected limb and their desires about what they would like their limb to be like. Perceptions were assessed for four separate aspects of body perception: size, shape, position, and definition (i.e., the sharpness of the limb's outline), and separately for sensations of what the limb looks like versus what it feels like. This rich dataset allowed the authors to investigate several novel aspects of body perception in CRPS.

Consistent with previous studies (e.g., Lewis et al. 2007), misperceptions of the limb were very common and took a wide range of forms. Distortions of size, shape, position, and definition were all reported by 70%–90% of patients, and 96% of patients reported at least one distortion. While many patients reported distortions in all modalities, multivariate analyses showed that patients clustered into three distinct sub‐groups based on the patterns of disturbances across modalities and between visual and felt experience. This suggests that body perception disturbance in CRPS is not a single thing which is present or absent, but a more complex constellation of experiences. The present exploratory study provides little direct insight into the underlying mechanisms distinguishing these sub‐groups. But future research should move from asking whether body perception disturbances are present to asking what kinds of disturbance occur, in what combinations, and what these reveal about the neurocognitive processes underlying body representation and pain.

Perhaps the most novel aspect of the study is the inclusion of questions about the desired state of the limb. More than 90% of patients indicated that they would desire to change the visual and felt properties of the affected limb. While this number is striking, it is also important to note that there was no non‐CRPS control group, so it is unclear how common such desires might be even in the non‐clinical population. It will be important in future research to obtain baseline data to provide context to understand how specifically this high level of desire for change is to CRPS.

One intriguing finding was that most patients expressed a desire for changes to the limb which were not exactly what they considered to be a ‘typical’ limb. It is intuitive to imagine that CRPS might be linked to a distorted feeling of the affected limb and that patients might therefore desire for the limb to feel normal. The present results, in contrast, suggest a much more complex relationship between perceived and desired body states. The body that CRPS patients want is not necessarily the body that they regard as normal.

Previous research has shown that allowing CRPS patients to manipulate their perceived limb using augmented reality can effectively reduce pain (Lewis et al. 2021). It is natural to interpret this as a normalisation of perceived body properties. The present results, however, suggest that the benefits of such procedures may result not from making the limb appear normal, but from making it appear abnormal in particular ways. More broadly, these results suggest that the body perception disturbances in CRPS should not be understood as deviations from a veridical representation of typical body representation. It remains unclear, however, what factors determine deviations of desired limb form from normality.

While exploratory, the study of Naylor et al. (2026) provides intriguing evidence that body perception disturbances in CRPS are ubiquitous and heterogeneous. By examining not only how patients perceive their affected limb but also how they wish it to be, the study opens new avenues for understanding disturbed body experience and its contribution to chronic pain. Future hypothesis‐driven studies can build on these findings to clarify both underlying mechanisms and therapeutic implications.

## Author Contributions

This commentary was written by Matthew Longo.

## Conflicts of Interest

The author declares no conflicts of interest.

## Linked Articles

This article is linked to Naylor et al. papers. To view this article, visit https://doi.org/10.1002/ejp.70401.

## References

[ejp70418-bib-0001] Lewis, J. S. , P. Kersten , C. S. McCabe , K. M. McPherson , and D. R. Blake . 2007. “Body Perception Disturbance: A Contribution to Pain in Complex Regional Pain Syndrome (CRPS).” Pain 133, no. 1: 111–119. 10.1016/j.pain.2007.03.013. 17509761

[ejp70418-bib-0002] Lewis, J. S. , R. Newport , G. Taylor , M. Smith , and C. S. McCabe . 2021. “Visual Illusions Modulate Body Perception Disturbance and Pain in Complex Regional Pain Syndrome: A Randomized Trial.” European Journal of Pain 25, no. 7: 1551–1563. 10.1002/ejp.1766. 33759278

[ejp70418-bib-0003] Naylor, C. E. , G. Buckingham , C. Lutteroth , and J. H. Bultitude . 2026. “Variations in Current and Desired Limb Perception in Complex Regional Pain Syndrome.” European Journal of Pain 30, no. 9: e70401. 10.1002/ejp.70401. PMC13651783 42853221

